# The *Research Fairness Initiative*: Filling a critical gap in global research ethics

**DOI:** 10.12688/gatesopenres.12884.1

**Published:** 2018-11-15

**Authors:** James V. Lavery, Carel IJsselmuiden

**Affiliations:** 1 Hubert Department of Global Health, Rollins School of Public Health and the Center for Ethics, Emory University, Atlanta, GA, 30322, USA; 2 Council for Health Research for Development, Geneva, Switzerland; 3 South African Research Ethics Training Initiative (SARETI), School of Applied Human Sciences, University of KwaZulu-Natal, Pietermaritzburg, South Africa

**Keywords:** Global Health, Research Partnerships, Research Ethics, Fairness

## Abstract

2017 marked the 70 ^th^ anniversary of the Nuremberg Code. The ethics of research with human beings has been shaped by the simplicity of its core logic, i.e., that the voluntary consent of research participants is sacrosanct and, when given, creates profound obligations of care and respect on the part of researchers. But there are other aspects of the global research enterprise that warrant more deliberate ethical scrutiny. One of these is the fairness of research collaborations and partnerships and the many practical challenges that make fair partnerships difficult to achieve. Corruption in governments and institutions, unequal access to research funding among researchers and research institutions, and enormous disparities in institutional capacity to support research partnerships are just some of the factors that present obstacles to fair partnerships between high income country (HIC) and low and middle income country (LMIC) partners, and within LMICs and HICs alike. Serious attention to these structural disparities, and the ways they shape the ethical character of the research enterprise, is long overdue. Achieving fairness in research partnerships is, in essence, a complex policy and management challenge. Against this backdrop, COHRED has developed and pilot-tested the *Research Fairness Initiative* (RFI) with several leading research institutions around the world. The RFI was designed as a tool for promoting self-reflection on, and public reporting of, institutional practices and policies related to research partnerships to create a continuous improvement process for research collaborations. Here, we report promising preliminary results of the RFI’s impact, including TDR-WHO’s recent publication of its first RFI report ***. ***The RFI provides a pragmatic strategy to explicitly address fairness in research partnerships as a fundamental requirement of the ethics of research.

## Background

2017 marked the 70 ^th^ anniversary of the Nuremberg Code ( Nuremberg Code, 1947). The ethics of research with human beings has been shaped by the simplicity of its core logic, i.e., that the voluntary consent of research participants is sacrosanct and, when given, creates profound obligations of care and respect on the part of researchers. It is a testament to the power of the Nuremberg Code, and the universal revulsion at the crimes that motivated it, that research ethics has remained intensively focused on the protection of individuals from potential harms that could arise as a result of their participation in research. There is no disagreement that this was and remains a necessary focus. But there are other aspects of the global research enterprise that warrant more deliberate ethical scrutiny. One of these is the fairness of research collaborations and partnerships ( Beran *et al*., 2017; Costello & Zumla, 2000; de Vries *et al*., 2015; Dodson, 2017; Parker & Kingori, 2016; Piotrowski & Melber, 2014; Shuchman *et al*., 2014; Zumla *et al*., 2010), and the many practical challenges that make fair partnerships difficult to achieve. Corruption in governments and institutions, unequal access to research funding among researchers and research institutions, and enormous disparities in institutional capacity to support research partnerships are just some of the factors that present obstacles to fair partnerships between high income country (HIC) and low and middle income country (LMIC) partners ( Parker & Kingori, 2016), and within LMICs and HICs alike ( de Noni *et al*., 2018). Serious attention to these structural disparities, and the ways they shape the ethical character of the research enterprise, is long overdue. It holds significant promise for reframing the ethics of research, and for illuminating rationales and pathways for greater investment in strong and sustainable research system capacity in all countries.

## Efforts to address obstacles to fairness in research partnerships

There have been many efforts to improve the fairness of research partnerships. Many have been driven by the commitments and actions of individual researchers in the design and management of their own collaborations, which tend not to be well publicized. Some have emerged as efforts to publicize ethically problematic imbalances in power, expectations and opportunities in research partnerships ( Zumla *et al*., 2010), and some have focused on specific aspects of fairness, such as systematic differences in opportunities to publish research results ( Matheka *et al*., 2014) and in resulting differences in opportunities for career advancement ( Nordling, 2014). Some negotiated agreements between research partners have been published ( Tierney *et al*., 2013), and research funders frequently include specific conditions of partnership in their investments ( Yarmoshuk *et al*., 2018), but there is typically no explicit mechanism by which the achievement of these conditions is adjudicated or reported.

A number of guidelines and frameworks have been developed and disseminated in efforts to direct more systematic changes in policy and practices ( Commission for Research Partnerships with Developing Countries, 2014; Canadian Coalition for Global Health Research, 2015; Institute for Development Research, 2012; Montreal Statement on Research Integrity, 2013) These efforts have had limited impact on the culture of research partnerships globally, as evidenced by the chronic and consistent nature of the critiques ( Beran *et al*., 2017). Although they effectively diagnose the problems associated with fair research partnerships, and provide useful taxonomies of general goals for improving practices, they are largely aspirational and lack sufficient detail and explicitness in their proposed strategies for achieving these goals.

Some initiatives have attempted to move beyond aspiration. For example, to increase the capacity of universities to negotiate fair collaboration agreements with industry, the UK government’s Intellectual Property Office produced the Lambert Toolkit, which provides tools and model agreements to facilitate fair and effective partnership agreements between university-based researchers and industry partners ( GOV.UK, 2017). Similarly, in an effort to improve the negotiation skills and contracting expertise of LMIC institutions, the Council on Health Research for Development (COHRED) developed the Fair Research Contracting suite of publications and tools ( Trust Project & COHRED, 2017). But although these initiatives explicitly aim to level the playing field to make fair agreements more likely, they are unable to undo the vast differences in wealth and power that frequently occur between major research organizations and less-well-resourced prospective partners.

In some cases, legal instruments have been introduced in an attempt to neutralize these power differentials. For example, the *Convention on Biological Diversity’s Nagoya Protocol on Access to Genetic Resources and the Fair and Equitable Sharing of Benefits Arising from their Utilization* provides a legal framework to guide the fair sharing of benefits arising from the use of genetic resources in research and other contexts ( Convention of Biological Diversity, 2014). Although such international legal instruments are important vehicles for raising awareness about various ethical hazards, they involve slow and cumbersome processes, are binding only on signatory countries, require substantial legal expertise to implement, and their corrective impact on research culture is difficult to gauge.

Achieving the ethical goal of fairness in research partnerships is, in essence, a complex policy and management challenge, made even more complicated by enormous variability in the nature of the studies and research programs, and in the specific contexts of the partnerships themselves. This raises three highly inter-dependent challenges. First, to elucidate and articulate the fundamental determinants of fairness in research partnerships. Second, to develop a methodology to promote and operationalize these determinants at a global level. And third, to demonstrate through the systematic collection of empirical evidence how their pursuit and achievement add value for participating organizations and the research enterprise more broadly.

## The *Research Fairness Initiative* (RFI)

Against this backdrop, COHRED has developed and pilot-tested the *Research Fairness Initiative* (RFI) ( COHRED, 2018) with several leading research institutions around the world ( Musolino *et al*., 2015). This process involved exploratory consultations with 32 public and private sector organizations in 15 countries ( COHRED, 2015a), followed by a global consultation in 2015, hosted by the Wellcome Trust ( COHRED, 2015b). Pilot-testing and implementation began in 2017 with a range of global research stakeholders, including the Special Programme for Research and Training in Tropical Diseases of the World Health Organization (WHO/TDR), Senegal’s Ministry of Higher Education, Research and Innovation in conjunction with three Senegalese research organizations and funders, the South African Department of Science and Technology, the Kenyan Medical Research Institute (KEMRI), and the Institute of Tropical Hygiene and Medicine in Portugal. Three institutional RFI Reports have now been published – WHO/TDR, Université Alioune Diop de Bambey, and the Instituto de Higiene e Medicina Tropical (IHMT), Universidade Nova de Lisboa ( IHMT, 2018) while several other institutions in Europe and Africa have started their reports.

The RFI was designed as a tool for promoting self-reflection on, and public reporting of, institutional practices and policies related to research partnerships ( COHRED, 2018). The RFI aims to create a continuous improvement process for research collaborations at four levels.

*Internally*, within participating institutions and organizations themselves, the RFI makes explicit and promotes the alignment of collaborative practices with organizational values, and aims to improve the quality and efficiency of research processes and the quality, cost-effectiveness and value of research partnerships for the RFI reporting organization itself.
*Externally*, the RFI provides organizations with a unique channel to communicate their commitment to fair partnership standards to partners and stakeholders, demonstrate responsible organizational, and corporate citizenship in R&D, and to enhance their trustworthiness as partners through transparency, a key determinant of lasting and productive research collaboration.
*Nationally (and regionally)*, aggregate analysis of RFI reporting rapidly highlights gaps and deficiencies in national research systems that can pose obstacles to fairness in research collaborations for all or many institutions at the same time. For example, the absence of a Material Transfer Agreement can be easily remedied and applied to all institutions, and relatively quickly improve the capacity of the institutions in the country concerned to negotiate fairer research relationships. Such aggregate analysis provides an ongoing and specific agenda for action by governments, development and research partners alike.
*Globally*, the RFI creates the means to build and share the first systematic global evidence-base for practices, policies, strategies, standards and benchmarks and their contribution to fair partnerships. Given that this topic concerns science collaboration, it is paradoxical that to date we know of no systematic learning or training opportunities on this key determinant of the success of research partnerships.

The RFI also represents a coherent extension of the logic of recent developments in community and stakeholder engagement (CSE) in research ( Lavery, 2018), which emphasize the critical importance of relationships and the ethical significance of acknowledging and addressing stakeholder interests in the context of research programs and projects, without obstructing or arbitrarily burdening the conduct of research ( King *et al*., 2014). To the extent that proponents can demonstrate the transferability of these integral aspects of CSE to research partnerships, there is great potential for synergies in logic, methods, strategies, and relevant empirical research.

## Early results from implementation of the RFI

We now have promising preliminary results from each of the RFI’s four intended levels of impact, described above. The leading example of the *internal* value of the RFI for research organizations is the TDR-WHO’s recent publication of its first RFI report ( TDR, 2018), which describes the internal process that the RFI provides guidance for:

*“The RFI provides a framework that allows an organization to take a step back and challenge itself to think about how its processes and approaches affect its partners. How do we select research priorities so they are in line with the needs of the country? Does our application process favour male applicants over women? How should benefits be shared and are contributions properly acknowledged? It is vital that we all continually ask ourselves questions like these.”* ( TDR, 2018, p. 1)

The *external* value of the RFI is reflected in the early experience in research institutions in Senegal, which were the first institutions to submit their institutional RFI reports. The institutions reflect a cross-section of research activity: a university with a rural development focus, the Alioune Diop University of Bambey; the largest and most successful privately funded HIV research and training institute in West Africa, Institut de Recherche en Santé, de Surveillance Epidémiologique et de Formation (IRESSEF); and a local funder of maternal and child health research in West Africa, Centre d’Excellence Africain pour la Santé de la Mère et de l’Enfant (CEA-SAMEF). The RFI reports of IRESSEF and CEA-SAMEF are in the process of final edits and will be published soon. These institutions produced their RFI reports in coordination with the Senegalese Ministry of Higher Education, Research and Innovation. Reviewing the draft reports allowed the ministry to see common gaps that put Senegalese institutions at a potential disadvantage when negotiating fair terms in collaborative research agreements. It is clear that this type of systematic reporting can significantly improve coordinated learning beyond the institutions themselves.

The potential *regional and global* impact of the RFI is illustrated by the experience of the Institute of Tropical Hygiene and Medicine in Portugal. In addition to any internal value for the organization, the completion of the RFI report generated an unexpected opportunity to coordinate strategy among the Ministers of Health of the Community of Portuguese Speaking Countries (CPLP), who recently decided unanimously to adopt the RFI as the instrument of choice to facilitate fair research collaborations between the CPLP countries ( CPLP, 2017). The RFI reporting process confirmed for the ministers that their institutions already have policies and practices that address many of the aspects of fairness covered in the RFI. Importantly, however, the RFI provided the process, and created the momentum, to bring the ministers together to consider the nature and quality of research collaborations between their countries from an international perspective. 

## Challenges ahead

Two issues are most likely to slow the speed of adoption of the RFI. First, there is a perception that the RFI will add uncompensated administrative burdens onto organizations. Second, some institutions have expressed concern that honest reporting and publication of ‘areas for improvement’ may reduce their competitiveness for partnership opportunities and external funding.

While any meaningful process of internal review and self-assessment carries an administrative burden for the organization, early experiences implementing the RFI have not emphasized such costs. The RFI indicators have been designed around commonly used data, and once the initial RFI report has been completed, the process requires reporting only of changes biennially. The RFI process has been viewed by participating organizations as a clear pathway to improve their research competitiveness, in addition to improving their own contributions to fair partnership practices. For research funders, the potential to measure the relationship between the quality of research partnerships and the impact of the research itself opens new space for program planning, design, management and evaluation in ways that could have a significant impact on the ethics and management of research programs. The RFI offers the conceptual architecture to support the development of such an empirical research program. And it also makes possible new opportunities for collaboration and integration with related initiatives, such as efforts to build an evidence-base for stakeholder engagement in science programs ( Lavery, 2018), and innovations in the way funders assess the quality and value of their research programs ( Lebel & McLean, 2018). 

Whether RFI reporting could compromise the competitiveness of research institutions, perhaps most importantly those in low and middle income countries, is an uncertainty inherent in any attempt to identify and highlight unfair research practices. To date, we have seen no evidence of such an effect. Instead, we have reports of how the internal review required by the RFI has exposed opportunities for relatively easy improvements. To be effective, the RFI will need to be responsive to the learning and improvement needs of any participating organizations that might be particularly vulnerable to this effect and offer support—perhaps through RFI “improvement partnerships” with high performing RFI institutions. These mechanisms will be facilitated by ongoing improvements in stakeholder representation in the governance and future development of the RFI that are currently underway.

Like Wikipedia, the quality and impact of the RFI will be determined by the scale and diversity of its contributors. A critical challenge is to accelerate the uptake of RFI reporting so that the RFI evidence-base ( COHRED, 2018b) increasingly reflects a broad range of organizations in various states of readiness for fair research partnership. As this process advances, we expect a continuous improvement in the ability of the RFI platform to support the learning and guidance necessary to establish fairness as a critical driver of institutional competitiveness and of ethical practice, beyond the important, but limited, focus of research ethics on the protection of research participants. The *Research Fairness Initiative* can help to fill a critical gap in the dominant research ethics paradigm by providing a pragmatic strategy to explicitly address fairness in research partnerships as a fundamental requirement of the ethics of research. It also provides the necessary infrastructure to develop a novel domain of empirical research that could provide badly-needed evidence to guide improvements in practice.

The key questions for scaling the RFI include: how to help institutions to find the balance between the costs and potential benefits associated with early adoption of the RFI; what sustainable benefits will low and middle income country institutions, in particular, realize from the initiative; and how can the RFI contribute to the evolution, and improvement, of ethics review of collaborative research and to strategies to improve research integrity ( Hudson, 2008). Reflections about fairness in research partnerships should no longer be relegated to the bar or the lunchroom. The RFI offers a strategy to guide these reflections within organizations and to share lessons and insights globally to address a critical gap in conventional research ethics.

## Disclaimer

The views expressed in this article are those of the authors. Publication in Gates Open Research does not imply endorsement by the Gates Foundation.
