# Supplementary material for: The Research Fairness Initiative: Filling a critical gap in global research ethics
Source: Gates Open Res. 2018 Nov 15;2:58. [Version 1] doi: 10.12688/gatesopenres.12884.1 (PMC6350408; doi:10.12688/gatesopenres.12884.1)
Supplement: Peer review history

# Peer review: Reviewer response for version 1

**DOI:** 10.21956/gatesopenres.13977.r26768

**Published:** 2018-12-12

**Authors:** Robert K. D. McLean, Tricia Wind

**Affiliations:** 1 Faculty of Medicine and Health Sciences, Stellenbosch University, Tygerberg, South Africa; 2 International Development Research Centre, Ontario, Canada

Thank you for the opportunity to review this well constructed letter, introducing and drawing attention to the importance of the RFI and the potential it has begun to present to the research ecosystem broadly. We present a series of suggestions we feel are necessary to consider before you finalize this letter.

 It would be very useful if the opening section provided a simple description of what a “research partnership” is. This is not so obvious to all readers and many will have particular frames of reference coming to this paper. For instance – partnerships between researchers on a single project, partnerships between faculties or universities on particular thematics, partnerships between proactive funders (as many foundations and philanthropies exhibit) and research teams receiving grants, partnerships between researchers and stakeholders, partnerships between foreign funders and recipient governments, etc. It would be helpful to articulate the parameters of the RFI view on partnerships early on in the letter.

 It would be helpful if the authors would explain two things about the RFI more clearly:

 a) What the RFI is:

 Is the RFI a checklist? A report format? Does it include a funding mechanism to support partnerships (p.5, top of second column)?

 What aspects of partnerships does it address? The letter refers to partnership equity issues variously as related to structural disparities, cultural issues, capacities, legal issues…does the RFI cover all those aspects?

 What is the unit of analysis for the RFI? It is organizations? And primarily research producing organizations? How does the RFI link with community and stakeholder involvement?

 Please help us to understand who fills out the RFI “report” and how they have the ability/knowledge to do so?  

 To whom do RFI reports go?

 b) The theory of change as to how the RFI will support more equitable partnerships.

 The RFI seems to be at a moment of seeing promising early uptake in a set of contexts (it’s not clear whether the experiences were connected with one another). This article gives helpful insight into these experiences. But what needs to happen in order to realize the national and global improvements that are outlined? Do the Initiative need a critical mass within certain countries, regions or across the globe? What would that entail?

 In what ways will the RFI support ongoing learning and improvement? Are the biennial reports the main mechanism through which the RFI supports change?

 Without knowing these details about the RFI, readers may not understand how the RFI will be an effective mechanism in improving equity in partnerships.

 The grounding of this letter in paving a way to more holistic examinations of ethics makes good sense.  However, we’re not sure that referencing the Nuremberg Code in the first paragraph is the best starting point for the topic at hand. The authors could instead outline a little further the points raised in the second paragraph about the problems of inequitable partnerships and how intractable they have been, because they are multifaceted. Alternatively, if they started with Nuremberg because they wish to connect it with racist aspects of inequitable research partnerships, then perhaps they could draw that out further. Racism is in part a power issue, research partnerships are also loaded with power dynamics that should be carefully minded.

 In addition, the authors might do well to embrace another impetus for the importance of the RFI. For better or for worse, funding agencies around the world are increasingly pushing for research partnerships across many strata. Sometimes this is couched in capacity building, sometimes it is a part of broad objectives for the internationalization of science, sometimes it is intended to drive research impact. It might be helpful for positioning the importance of this initiative to acknowledge the many reasons partnerships are becoming more and more common. It could then be acknowledged that many enter partnerships not on their own desire, but to access funder programming. This immediately implies a power dynamic that may precipitate ethical concerns. For example, in a recent international review of funders efforts to promote KT, “linkage and exchange” between researchers and users of research is the emergent trend for promoting knowledge translation.

 The conclusion of the letter seems to require a review for consistency of logic. Two concerns are stated about the RFI moving forward. But then, the defence/response to concern 1 is presenting the anti-thesis of concern 2. This becomes a little cyclical for the reader and could be clarified with a simple edit of the logic. We mean the sentence in para 2 of the conclusion which begins: “The RFI process has been viewed participating organizations as a clear pathway to improve their research competitiveness….”  We also note that it might be good to qualify how this claim was reached.

  Editorial, clarification, and balance issues:

 Abstract – it is likely stretching too far to claim this is a reflection of the “RFI’s impact” in the second last sentence.  Maybe “RFI’s potential”.

 Background final para – in the final paragraph please clarify  “their pursuit”.  Not partnerships we assume?

 RFI section – final sentence needs further clarification or explanation. Why is there great potential for synergies between CSE and RFI?

 Early results section para 2 - the authors might wish to step down a little on the final sentence.  Is it really clear that this means “significant coordinated learning beyond the institutions themselves”?  If so, please explain how this is clear? To us it lays excellent groundwork but would probably require a significant additional effort and perhaps resources to facilitate open and fair coordination across agencies.

 Early results section final para – It is not entirely clear how ministers used the RFI to “understand the nature and quality of research partnerships between their countries from an international perspective”. Could this be explained or exemplified so it is clear? It sounds like a remarkable achievement, please show us how it was reached.

 Challenges ahead section para 4  - Are the authors stretching ahead of current success with the Wikipedia metaphor? We see who these relate to objectives 3 and 4 of the RFI, but the first successes to be pushed for and celebrated once achieved, are about “internal” and “external” organizational use. Saying the RFI is only as good as its scale is big, seems to understate early wins that may result around these perhaps equally important local goals?

We have read this submission. We believe that we have an appropriate level of expertise to confirm that it is of an acceptable scientific standard.

# Peer review: Reviewer response for version 1

**DOI:** 10.21956/gatesopenres.13977.r26767

**Published:** 2018-11-22

**Authors:** Jantina de Vries

**Affiliations:** 1 Department of Medicine, University of Cape Town, Cape Town, South Africa

Thank you for this paper which I enjoyed reading. One challenge in the paper is that it is not entirely clear what work you hope to do in the paper. Is this generally about presenting the RFI (and if so, how does this paper relate to the Lancet paper by Musolino et al?); to present some early use case scenarios; or to present a more critical piece about the challenges towards implementation. It may be good to add a few sentences at the end of your introduction describing more clearly the aim of the manuscript, to help orient the reader. Other comments are:

- On pg. 4 of 6, before detailing what the RFI aims to do, it would be good to add a short description of what the RFI is (e.g. is it a list of questions that need to be answered systematically?). This includes some mention of the formalisation of the RFI process – e.g. that COHRED vets the reports, and that ‘the process requires reporting only of changes biennially’. This leads me to wonder whether the RFI is kind of like an accreditation awarded by COHRED to organisations? At other places you speak about ‘the RFI platform’ and the ‘RFI evidence base’ and it would be good to give the reader some indication of what the RFI is;
- Overall, in the manuscript it is not entirely clear what the basis is for the observations you make in the section ‘early results from the implementation of the RFI’. e.g. when you make statements like ‘participating institutions view’ the RFI as X, then what is the basis for that claim? Did you speak with them at a meeting, did they write this in a reflection on the use of the RFI, or do you have another basis for this claim (e.g. your own observations and insights or perhaps interviews?). You describe your insights as ‘results’ but are they really that?;
- You say that “Reviewing the draft reports allowed the ministry to see common gaps that put Senegalese institutions at a potential disadvantage when negotiating fair terms in collaborative research agreements.” It would be good to embellish that observation with an example of ‘a common gap’ that was identified in this way, and any action that has been taken or that could be taken by the Senegalese ministry to ensure greater fairness in collaboration going forward;
- On pg 4 of 6 you also observe that “the completion of the RFI report generated an unexpected opportunity to coordinate strategy” -  how did the *completion* of the RFI lead to this opportunity? Did the RFI ‘bring ministers together’? Again, slightly greater elaboration of the example would help the reader understand exactly why the RFI was useful or helpful and what it achieved in this example (if, as the phrasing suggests, it was the RFI directly that had this effect of harmonisation between countries then that would be quite remarkable and worth mentioning)
- The sentence “The RFI process has been viewed by participating organizations as a clear pathway to improve their research competitiveness” on pg 5 of 6 also raises some questions. How can the index, which set out to promote self-reflection and identify institutional policies related to partnerships, improve research competitiveness of institutions? (Or: why and how did the participating organisations perceive it to have that effect). Note that in the following paragraph you ask ‘whether the RFI could compromise competitiveness’ – which is the complete opposite of the view of participating organisations. Which concern is greater and how would the RFI lead to either of these effects?
- That you have seen no evidence of particular effects (you mention costs in terms of administrative burden and reduced competitiveness as examples) of course does not mean that the effect may not be there. If I understand your paper correctly, the RFI has been used by several organisations in three distinct initiatives and in this paper you offer a soft assessment of the experiences of those organisations. Your assessment draws on the text published in those reports (WHO/TDR example) and on your observations and you have not provided the basis for that learning. Yet you seem to diminish these concerns in the way you describe them on pg 5 of 6 and you could perhaps consider taking out those particular phrases (‘we have seen no evidence of X’);
- Everywhere but in the before-last paragraph you refer to ‘the RFI’ but in that paragraph you give the full name in italics which is a bit odd. Perhaps just change for RFI there too.

- The sentence “the RFI can help to fill a critical gap in the dominant research ethics paradigm by providing a pragmatic strategy to explicitly address fairness in research partnerships as a fundamental requirement of the ethics of research.” is a bit odd. Does it ‘fill a gap’ in the research ethics paradigm (presuming that you mean the paradigm you started your paper with) or rather does is complement that particular paradigm. Does the RFI *address* fairness or does it offer a tool to map collaborative practices and identify particularly fair and unfair practices that can then be addressed through other means? And in the latter part of that sentence, do you mean to say that you think that ‘fairness in research partnerships’ needs to become/be emphasized as a fundamental requirement of the ethics of research?

I have read this submission. I believe that I have an appropriate level of expertise to confirm that it is of an acceptable scientific standard.
